# Метформин и злокачественные новообразования: возможный механизм противоопухолевого действия и перспективы использования в практике

**DOI:** 10.14341/probl13097

**Published:** 2022-07-14

**Authors:** К. О. Кузнецов, Э. Р. Сафина, Д. В. Гаймакова, Я. С. Фролова, И. Ю. Оганесян, А. Г. Садертдинова, К. А. Назмиева, А. Х. Исламгулов, А. Р. Каримова, А. М. Галимова, Э. В. Ризванова

**Affiliations:** Российский национальный исследовательский медицинский университет им. Н.И. Пирогова; Башкирский государственный медицинский университет; Башкирский государственный медицинский университет; Первый Московский государственный медицинский университет им. И.М. Сеченова; Первый Московский государственный медицинский университет им. И.М. Сеченова; Башкирский государственный медицинский университет; Башкирский государственный медицинский университет; Башкирский государственный медицинский университет; Башкирский государственный медицинский университет; Башкирский государственный медицинский университет; Башкирский государственный медицинский университет

**Keywords:** сахарный диабет, метформин, злокачественные новообразования, рак, механизм действия

## Abstract

Метформин является противодиабетическим препаратом первой линии для лечения сахарного диабета 2 типа (СД2); его молекулярной мишенью является АМФ-активируемая протеинкиназа (АМФК), которая участвует во многих метаболических процессах. Метформин не только снижает уровень глюкозы в крови и улучшает чувствительность к инсулину, но также ингибирует липолиз и снижает сердечно-сосудистый риск у пациентов с СД2. В последние годы доказано, что метформин замедляет процесс старения, стимулирует рост волос, устраняет когнитивные нарушения, а также обладает противоопухолевым эффектом. Большинство фундаментальных исследований показало, что метформин ингибирует рост опухолевых клеток и способствует клеточному апоптозу, в то время как клинические исследования показывают противоречивые результаты. Такое несоответствие можно объяснить разницей в концентрации метформина между фундаментальными и клиническими исследованиями. Максимальная суточная доза метформина для пациентов с СД2 составляет 2500 мг/сут, а доза, использованная в фундаментальных исследованиях, была намного выше. Метформин непосредственно активирует сигнальный путь АМФК, ингибирует выработку активных форм кислорода, индуцирует активацию mTORC1, ингибирует циклин D1, что приводит к снижению риска возникновения и развития злокачественных новообразований. Кроме того, метформин косвенно ингибирует рост опухоли, пролиферацию, инвазию и метастазирование путем снижения концентрации глюкозы в крови, улучшения чувствительности тканей к инсулину, а также путем уменьшения воспаления и влияния на микроокружение опухоли. Гликолиз играет важную роль в энергетическом обмене опухолей, а метформин способен оказывать на него ингибирующее влияние. В настоящее время исследования механизма противоопухолевых эффектов метформина становятся все более обширными и углубленными, однако по-прежнему остаются некоторые противоречия.

## Abstract (en)

Metformin is a first-line antidiabetic drug for the treatment of type 2 diabetes mellitus (DM2); its molecular target is AMP-activated protein kinase (AMPK), which is involved in many metabolic processes. Metformin not only reduces blood glucose levels and improves insulin sensitivity, but also inhibits lipolysis and reduces cardiovascular risk in patients with DM2. In recent years, it has been proven that metformin slows down the aging process, stimulates hair growth, eliminates cognitive impairment, and also has an antitumor effect. Most basic studies have shown that metformin inhibits the growth of tumor cells and promotes cellular apoptosis, while clinical studies show contradictory results. This discrepancy can be explained by the difference in the concentration of metformin between basic and clinical studies. The maximum daily dose of metformin for patients with DM2 is 2500 mg / day, and the dose used in basic research was much higher. Metformin directly activates the AMPK signaling pathway, inhibits the production of reactive oxygen species, induces the activation of mTORC1, inhibits cyclin D1, which leads to a reduction in the risk of the occurrence and development of malignant neoplasms. In addition, metformin indirectly inhibits tumor growth, proliferation, invasion and metastasis by reducing the concentration of glucose in the blood, insulin resistance, as well as by reducing inflammation and affecting the tumor microenvironment. Glycolysis plays an important role in the energy metabolism of tumors, and metformin is able to have an inhibitory effect on it. Currently, studies of the mechanism of antitumor effects of metformin are becoming more extensive and in-depth, but there are still some contradictions.

## ВВЕДЕНИЕ

В XXI в. неинфекционные заболевания, включая злокачественные новообразования, являются основными причинами смерти во всем мире [1]. Согласно последним глобальным статистическим данным о раке, в 2020 г. во всем мире было зарегистрировано 19,29 млн новых случаев рака и 9,96 млн смертей, связанных с раком. Рак молочной железы и рак легких являются наиболее распространенными видами рака во всем мире, за которыми следуют колоректальный рак (10,0%), рак предстательной железы (7,3%) и рак желудка (5,6%). Рак легких является основной причиной смерти от онкологических заболеваний, за которым следуют колоректальный рак (9,4%), рак печени (8,3%), желудка (7,7%) и молочной железы (6,9%) [2].

Сахарный диабет 2 типа (СД2) представляет собой метаболическое заболевание, характеризующееся снижением чувствительности тканей к инсулину и его секреции, что приводит к гипергликемии. Распространенность СД2 ежегодно растет, он находится на 6-м месте среди причин смерти во всем мире [3]. Эпидемиологические исследования показали, что пациенты с СД2 имеют повышенный риск развития рака печени, поджелудочной железы, почек, молочной железы и других злокачественных новообразований [4]. Повышенная экспрессия инсулиноподобного фактора роста (ИФР), развивающаяся на фоне гипергликемии и инсулинорезистентности, стимулирует развитие злокачественных опухолей. Пролиферативные процессы во внутренних органах также увеличивают риск развития рака [5]. В глобальном плане СД2 связан с повышенным риском развития онкологических заболеваний, что вызывает интерес к определению эпидемиологических и биологических связей между СД2 и злокачественными опухолями [3].

Метформин — препарат, препятствующий повышению гликемии, который является безопасным, эффективным и недорогим. Нарастающее количество исследований показывает, что метформин может улучшать липидный обмен, способствовать снижению массы тела, снижать частоту сердечно-сосудистых заболеваний, замедлять снижение когнитивных функций, а также снижать риск развития деменции [6–8]. Кроме того, фундаментальные и клинические исследования показали, что метформин обладает противоопухолевым действием и повышает чувствительность к химиотерапии [9]. Однако текущие исследования показали, что влияние метформина на различные опухоли является спорным, а механизм его противоопухолевого действия не совсем ясен. В настоящем обзоре рассмотрены и проанализированы влияние метформина на различные злокачественные новообразования, а также возможный механизм такого влияния.

## ВЛИЯНИЕ МЕТФОРМИНА НА ЗЛОКАЧЕСТВЕННЫЕ НОВООБРАЗОВАНИЯ

## Рак молочной железы

Рак молочной железы (РМЖ), по оценкам, был наиболее распространенным видом рака в 2020 г., число новых случаев составило 2,26 млн (11,7%) [2]. Хорошо известно, что СД является ключевым фактором риска развития РМЖ за счет изменения метаболизма эстрогенов и функции рецепторов, тем самым увеличивая риск развития и прогрессирования опухоли [10]. Пролиферация и распространение РМЖ тесно связаны с клеточным метаболизмом глюкозы, учитывая, что глюкоза является важным клеточным метаболическим субстратом и передача сигналов инсулина оказывает митогенное действие [11]. Исследования показали, что у женщин с СД, принимающих метформин, наблюдалась более низкая заболеваемость РМЖ [12], чем у женщин, которые использовали другие противодиабетические препараты. В дальнейшем растущее число клинических и фундаментальных исследований показало, что метформин снижает заболеваемость, метастазирование и частоту рецидивов РМЖу женщин [13][14]. L. Chen и соавт. провели ретроспективное когортное исследование, которое включало 14 766 женщин с недавно диагностированным РМЖ I и II стадии, 791 из них имели метастазы в других органах, 627 испытали рецидив заболевания, а 237 умерли от рака молочной железы. Результаты показали, что использование метформина (n=2558) снижает риск метастазирования, рецидива и смерти от РМЖ на 28, 31 и 49% соответственно [15]. J.C. Wang и соавт. создали мышиную модель метастатического РМЖ и проводили оценку скорости метастазирования в легкие и чувствительности к химиотерапии у мышей, получавших метформин, и в контрольной группе. Авторы пришли к выводу, что метформин снижает скорость легочного метастазирования первичных опухолей и повышает чувствительность к химиотерапии, это может быть обусловлено сосудистыми эффектами метформина [16]. На сегодняшний день большинство исследований метформина при РМЖ было сосредоточено на пациентах с СД [17][18], однако рандомизированное двойное слепое плацебо-контролируемое исследование II фазы, проведенное в 2012 г. при участии пациентов с РМЖ без СД, пришло к другим выводам относительно действия метформина. В исследование включались пациентки с РМЖ I–IIa стадии без СД; все они готовились к плановому хирургическому вмешательству и получали либо метформин, либо плацебо в течение 4 нед до операции [19]. Результаты показали, что метформин до операции не оказывал существенного влияния на общую пролиферацию РМЖ, но при этом оказывал ингибирующее влияние на пролиферацию у женщин с повышенным индексом инсулинорезистентности (HOMA), в то время как противоположная тенденция наблюдалась у женщин с нормальной чувствительностью к инсулину [19]. В этом исследовании метформин использовался до операции в течение относительно короткого периода времени, а на прогрессирование РМЖ влияли такие факторы, как эмоциональное состояние и гормональный статус. Для подтверждения этой точки зрения необходимы дальнейшие исследования.

Тройной негативный рак молочной железы (ТНРМЖ) является подтипом с самой низкой пятилетней выживаемостью. ТНРМЖ высокоинвазивен и легко метастазирует, а терапевтический эффект против него незначительный [20]. В 2013 г. R. Wahdan-Alaswad и соавт. продемонстрировали, что метформин способствует апоптозу и некрозу клеток РМЖ, особенно клеток ТНРМЖ, за счет снижения экспрессии инсулиноподобного фактора роста-1 (ИФР-1), однако этот эффект ингибировался при повышении концентрации глюкозы [21]. В дальнейшем авторы стремились исследовать механизм метформин-индуцированного ингибирования ТНРМЖ и доказали, что метформин индуцировал гибель клеток ТНРМЖ и блокировал деление стволовых клеток посредством активации микроРНК-193b, которая подавляет синтазу жирных кислот [22]. R. Wahdan-Alaswad и соавт. также изучали влияние метформина на мезенхимальный стволовой ТНРМЖ с низкой экспрессией клаудина (MSL/CL). Авторы обнаружили, что метформин ингибирует возникновение РМЖ MSL/CL путем подавления сигнальной функции трансформирующего фактора роста β (ТФР-β) через ингибирование Smad2 и Smad3 в клеточных линиях MSL/CL, а также ослабляет индуцированный ТФР-β эпителиально-мезенхимальный переход за счет ингибирования Snail-белков в клеточных линиях MSL/CL, тем самым снижая миграцию и инвазию опухоли [23]. Два года спустя авторы подтвердили, что ингибирование пролиферации ТНРМЖ метформином может быть связано с ингибированием пути синтеза холестерина [24]. Кроме того, B. Liu и соавт. изучали влияние метформина на устойчивые к трастузумабу клетки РМЖ. Результаты показали, что метформин ингибировал пролиферацию устойчивых к трастузумабу клеток РМЖ путем инактивации erbB3 и ИФР-1, а также инактивируя Src-киназу и/или путь PI-3K/Akt [25]. Однако другие исследователи придерживаются иной точки зрения. В 2019 г. H. Chen и соавт. провели ретроспективное многоцентровое исследование, направленное на изучение влияния противодиабетических препаратов на риск развития различных подтипов РМЖ [26]. В исследование были включены 1992 пациентки с ER+/HER2-, 324с ER+/HER2+, 1446 пациенток с ТНРМЖ и 578 с HER2+. По результатам исследования было выявлено, что женщины с СД2 имели более высокий риск развития ТНРМЖ (на 38% выше, чем в общей популяции), а также длительность приема метформина имела положительную корреляцию с риском возникновения ТНРМЖ [26]. Тем не менее в этом исследовании в основном оценивался риск развития различных типов РМЖ у пациенток с СД, и влияние метформина на этот риск было низким. Нет убедительных доказательств того, что метформин связан с повышенным риском ТНРМЖ.

В рамках рандомизированного клинического исследования T.Y. Semiglazova и соавт. показали, что добавление метформина (в дозе 850 мг 2 раза в день) к неоадъювантной гормонотерапии торемифеном на протяжении 4 мес значимо подавляло пролиферативную активность опухоли — суррогатного маркера улучшения выживаемости у больных гормоночувствительным РМЖ без СД в анамнезе. Снижение маркера Ki-67 наблюдалось в 4,2 раза чаще в группе комбинированной терапии метформином и торемифеном по сравнению с только гормонотерапией торемифеном (95% ДИ 1,04–17,1; р=0,043). Также была выявлена значимая корреляция между динамикой снижения индекса Ki-67 в опухоли и индексом массы тела выше нормы (р=0,015) [27].

В настоящее время многие исследования показали, что метформин может снижать риск развития РМЖ у пациенток с СД. Согласно результатам ряда работ когортного и «случай-контроль» типа, применение метформина на фоне комплексного лечения больных РМЖ приводит к увеличению безрецидивной и общей выживаемости [28]. Тем не менее имеется мало исследований влияния метформина на пациенток с РМЖ без СД и пациенток с различными типами РМЖ, а также имеются некоторые противоречия; таким образом, необходимы дальнейшие клинические исследования.

В настоящее время в НИИ онкологии им. Н.Н. Петрова проводятся 2 рандомизированных проспективных исследования, где оценивается эффективность неоадъювантной химиотерапии или гормонотерапии у больных с местно-распространенным РМЖ [28]. Целью исследований является проверка гипотезы о влиянии метформина на эффективность неоадъювантной химиотерапии и гормонотерапии у пациенток РМЖ.

## Рак легких

Рак легких (РЛ) находится на 1-м месте в структуре смертности от онкологических заболеваний во всем мире [2]. Частота рецидивов выше у пациентов с СД, а ответ на терапию хуже, чем у пациентов с РЛ без СД [29]. Метформин исследовался в течение многих лет в качестве средства для адъювантного лечения рака легких. Предыдущие исследования показали, что метформин улучшает выживаемость пациентов с немелкоклеточным раком легкого (НМРЛ) и мелкоклеточным раком легкого (МРЛ) [29]. S. Zeng и соавт. провели метаанализ, в котором проанализировали 10 клинических исследований и 4397 пациентов. Результаты показали, что терапия метформином значительно улучшала выживаемость пациентов с РЛ. Авторы определили общую выживаемость (ОВ) как 0,78 для пациентов с НМРЛ и 0,51 для пациентов с МРЛ на фоне проводимой терапии [30]. S. Brancher и соавт. проанализировали связь между использованием метформина до и после постановки диагноза РЛ и общей выживаемостью, а также со специфической выживаемостью при РЛ [31]. Результаты показали, что использование метформина до постановки диагноза было связано с увеличением специфической выживаемости у пациентов как с плоскоклеточным РЛ, так и с регионарной плоскоклеточной карциномой. У всех пациентов с РЛ использование метформина после постановки диагноза также приводило к увеличению специфической выживаемости. Аналогичные результаты были получены для ОВ [31]. В рандомизированном клиническом исследовании, проведенном H. Skinner и соавт., было показано, что добавление метформина к химиолучевой терапии хорошо переносилось, но не улучшало выживаемость среди пациентов с неоперабельной стадией III НМРЛ [32]. Как клинические, так и эпидемиологические данные свидетельствуют о том, что метформин снижает заболеваемость РЛ. Исследования in vitro показали, что метформин ингибирует пролиферацию и инвазию опухолевых клеток путем активации PP2A, а он, в свою очередь, ингибирует активацию опухолеобразующих белков Bax, Myc и Act через каталитическое дефосфорилирование [33]. Клинические и фундаментальные исследования показали, что метформин оказывает протективное действие при РЛ, что открывает возможности для разработки нового метода лечения.

На сегодняшний день протективное действие метформина при РЛ является наиболее доказанным, т.к. многие исследования учитывали различные типы, стадии и лекарственную устойчивость РЛ. Несколько лет назад ряд больниц по всему миру начал применять метформин у пациентов с РЛ, что показало определенную эффективность. Поэтому после исключения противопоказаний метформин, как ожидается, будет регулярно использоваться в качестве вспомогательной терапии у пациентов с РЛ.

## Колоректальный рак

Уровень заболеваемости колоректальным раком занимает 3-е (более 1,93 млн новых случаев), а по смертности — 2-е место среди всех злокачественных новообразований (935 тыс. смертей) [2].

Появляется все больше доказательств того, что пациенты с СД2 имеют более высокий риск развития колоректального рака и смерти, связанной с ним [34]. Метформин может уменьшать размер опухоли и ингибировать его пролиферацию. Когортное исследование с участием 47 351 пациентов с СД продемонстрировало отрицательную корреляцию между длительностью применения метформина и риском развития колоректального рака, особенно у мужчин [35]. T. Higurashi и соавт. провели однолетнее клиническое исследование с целью оценки безопасности и эффективности метформина при спорадическом колоректальном раке у пациентов с высоким риском рецидива. Результаты показали, что низкие дозы метформина снижают частоту и количество метахронных аденом или полипов после полипэктомии и метформин играет потенциальную роль в химиопрофилактике колоректального рака [36]. Недавно было высказано предположение, что метформин может быть полезен для усиления терапевтического эффекта лучевой терапии у пациентов с раком прямой кишки [37]. Кроме того, исследователи также культивировали три различные линии опухолевых клеток (SW480, HT29, HCT116) с метформином на фоне подачи излучения. Авторы отметили, что метформин усиливает реакцию опухолей на излучение in vivo и in vitro [37]. Это исследование проводилось на клеточных и мышиных моделях, поэтому для проверки данного явления необходимы дальнейшие клинические обсервационные исследования. У пациентов с метастатическим колоректальным раком ОВ по-прежнему остается на низком уровне, особенно при наличии мутации гена KRAS, несмотря на проведение химиотерапии иринотеканом или оксалиплатином в сочетании с ингибиторами рецептора эпидермального фактора роста (EGFR). Аномальная передача сигналов KRAS играет решающую роль в пролиферации опухолевых клеток и обычно связана с неблагоприятным прогнозом и устойчивостью к терапии ингибиторами EGFR. Недавно китайские исследователи указали, что по сравнению с другими противодиабетическими препаратами в сочетании со стандартным лечением метформин избирательно ингибирует рост метастатического колоректального рака с мутацией в гене KRAS у пациентов с СД. Среднее время выживаемости в этой группе пациентов было продлено на 37,8 месяца. Это исследование определяет возможность использования метформина для лечения пациентов с метастатическим колоректальным раком с мутациями в гене KRAS [38].

На сегодняшний день существует большое количество исследований, посвященных изучению влияния метформина на колоректальный рак, начиная от предраковых поражений до прогрессирующего колоректального рака, и большинство из них подтверждает протективное действие метформина, однако некоторые авторы имеют противоположную точку зрения.

## Рак предстательной железы

Рак предстательной железы (РПЖ) находился на 2-м месте по заболеваемости у мужчин среди онкологических заболеваний в 2020 г., было выявлено 1,4 млн новых случаев и 375 тыс. смертей во всем мире [2]. Влияние метформина на риск возникновения РПЖ остается спорным. Метаанализ показал, что более низкая заболеваемость РПЖ у пациентов с СД2 может быть связана со снижением уровня тестостерона при отсутствии должного гликемического контроля [39]. Лечение СД2 метформином может обратить вспять условия, которые снижают уровень андрогенов, в результате чего рост, развитие и пролиферация опухоли могут усилиться. Недавнее ретроспективное исследование также показало, что терапия метформином повышает риск развития РПЖ [40]. Тем не менее другие исследования показали, что метформин снижает риск развития РПЖ. В 2013 г. D. Margel и соавт. сравнивали пациентов с недавно диагностированным СД в возрасте 66 лет и старше (в промежуток с января 1997 г. по 31 марта 2008 г.) с пациентами, у которых были диагностированы СД и РПЖ. Результаты показали, что терапия метформином снижает смертность как от СД, так и от РПЖ [41]. Применение метформина также снижало риск развития РПЖ у мужчин с СД2 в Тайване по результатам ретроспективного когортного исследования [42].

Андрогенный рецептор (АР) является значимым пусковым фактором в развитии РПЖ; андрогенная депривационная терапия (АДТ) — основной метод лечения прогрессирующего РПЖ, а снижение экспрессии АР считается профилактической стратегией для предупреждения рецидивов РПЖ [43]. Метформин способен снижать экспрессию АР путем усиления активности комплекса трансляционных регуляторов Midline-1 (MID1) и тем самым повышать эффективность АДТ. Кроме того, метформин может ингибировать сигнальные пути АР, снижая уровни мРНК АР [44]. Эти исследования подтверждают потенциальные эффекты метформина в сочетании с АДТ.

В настоящее время эффект метформина на РПЖ все еще неясен и может быть связан с его патогенезом. Поэтому не рекомендуется применять метформин у пациентов с РПЖ без СД, в то время как метформин может регулярно использоваться у пациентов с СД2 и РПЖ после исключения противопоказаний.

## Рак желудка

Рак желудка (РЖ), по оценкам, находится на 5-м месте по распространенности и на 4-м месте по смертности среди онкологических заболеваний [2]. Эпидемиологические исследования о взаимосвязи между СД и риском развития РЖ неубедительны, а их результаты противоречивы [45]. Тем не менее исследования показали, что риск развития РЖ у пациентов с СД2, получавших метформин, ниже, чем у пациентов, не принимающих его [43]. C.H. Tseng показал, что метформин значительно снижает риск развития РЖ, особенно если срок его применения составляет более 2 лет [46]. J. Kim и соавт. провели ретроспективное исследование, целью которого явилось выявление корреляционных связей между использованием метформина и риском развития РЖ у пациентов с СД. Пациенты в возрасте 40–80 лет были разделены на три группы: больные СД, применяющие метформин; больные СД, не применяющие метформин; пациенты, не страдающие СД. Среднее время наблюдения составило 12,7 года. Результаты показали, что заболеваемость РЖ была выше у пациентов, которые не принимали метформин, а самый низкий показатель заболеваемости был отмечен у пациентов без СД, которые принимали метформин. Наконец, авторы отметили, что пациенты с СД, которые использовали метформин, имели более низкий риск развития РЖ [47]. Клинические исследования отражают тот факт, что метформин снижает риск развития РЖ, однако отсутствует информация, оказывает ли метформин прямое противоопухолевое действие. Поэтому создается необходимость в изучении прямого влияния метформина на РЖ, а также его молекулярного механизма. Некоторые исследования показали, что метформин ингибирует пролиферацию и метастазирование клеток РЖ, а также способствует их апоптозу. G. Han и соавт. использовали метформин для лечения клеточных линий РЖ с разной дифференцировкой (MKN-28, SGC-7901, BGC-823 и незлокачественная линия GES-1). Результаты показали, что лечение метформином избирательно индуцировало апоптоз в низкодифференцированных клетках [48].

Как у пациентов с СД, так и без него метформин показал протективное действие при РЖ, что свидетельствует о наличии других механизмов действия, помимо препятствия повышению гликемии. При неэффективности стандартной терапии РЖ можно рассмотреть вопрос о включении метформина в качестве адъювантной терапии.

## Рак печени

Рак печени (РП) находится на 6-м месте по распространенности и на 3-м месте по смертности среди онкологических заболеваний [2]. СД на сегодняшний день считается независимым фактором риска развития РП и, как было показано, увеличивает риск развития РП даже у пациентов, не инфицированных вирусом гепатитов B и C [49]. Проспективное когортное исследование, проведенное M.S. Lee и соавт. в Тайване, показало, что метформин снижает риск первичного рака печени по сравнению спациентами, принимающими другую противодиабетическую терапию [50]. Также было доказано, что метформин в сочетании с лучевой терапией увеличивает ОВ пациентов с первичным РП [51]. Все предыдущие исследования показали значительное снижение заболеваемости гепатоцеллюлярной карциномой у пациентов с СД2, получавших метформин. Однако метформин практически не влияет на профилактику гепатоцеллюлярной карциномы у пациентов без СД. R.C. Shankaraiah и соавт. использовали тетрахлорметан (CCl4) на трансгенных мышах с чрезмерной экспрессией микроРНК-221 с целью создания модели РП на фоне цирроза [52]. Мышам вводили метформин в дозе 250 мг/кг/сут (что эквивалентно стандартной дозе 1500 мг у пациентов с СД). Результаты показали, что метформин улучшал функцию печени мышей, ингибировал активацию печеночных звездчатых клеток, уменьшал выраженность фиброза печени, снижал накопление липидов в гепатоцитах, задерживал прогрессирование цирроза и предотвращал развитие CCl4-индуцированного первичного рака печени у трансгенных мышей. Данное исследование обосновывает применение метформина у пациентов с циррозом печени, независимо от наличия СД [52].

На сегодняшний день существует мало противоречий относительно протективного эффекта метформина при РП, однако большинство клинических исследований было проведено на пациентах с СД и лишь небольшое количество — у пациентов без СД. Поэтому рутинное применение метформина у пациентов с РП без СД не рекомендуется, необходимо проведение дополнительных клинических исследований.

Помимо распространенных злокачественных новообразований, метформин оказывает протективное действие на другие опухоли, такие как рак поджелудочной железы, рак мочевого пузыря, рак желчевыводящих путей, рак почек и рак яичников [53].

C. Coyle и соавт. провели метаанализ, который включал 7670 статей, из них авторы выявили 27 подходящих исследований, включающих 24 178 участников. У пациентов с колоректальным раком на ранней стадии применение метформина было связано со значительным преимуществом во всех исходах. Для мужчин с ранней стадией РПЖ метформин также показывал улучшение терапевтического эффекта, однако между исследованиями наблюдалась значительная неоднородность. Данные свидетельствуют о том, что у пациентов с РПЖ, получавших радикальную лучевую терапию, метформин оказывает более значимый положительный эффект. При РМЖ и уротелиальном раке не было выявлено существенных преимуществ. Отсутствовали достаточные данные для проведения анализа влияния дозы и продолжительности применения метформина [54].

В.Н. Анисимов и соавт. оценивали результаты доклинических испытаний антиканцерогенных и противоопухолевых свойств бигуанидов. В экспериментах было использовано более 20 экспериментальных моделей канцерогенеза, включая модели спонтанного, химического, вирусного, радиационного, а также вызванного особенностями диеты и генетическими модификациями канцерогенеза. Антиканцерогенный эффект бигуанидов был изучен в отношении 17 органов-мишеней у животных 3 видов, включая 25 линий и разводок мышей, 4 линии крыс и 1 разводку хомячков при их введении разными способами и в разных дозах. В большинстве случаев (86%) бигуаниды оказывали угнетающее влияние на канцерогенез. В 14% случаев тормозящий эффект препаратов не был выявлен. Важно подчеркнуть, что ни в одном из исследований не наблюдалось стимуляции канцерогенеза антидиабетическими бигуанидами [55].

Таким образом, большинство клинических исследований подтвердило тот факт, что метформин может ингибировать возникновение и развитие опухолей. Тем не менее S.P. Srivastava и соавт. считают, что предположения о противоопухолевой активности метформина преувеличены [56]. T.K. Oh и соавт. провели общенациональное выборочное когортное исследование с целью анализа взаимосвязи между использованием метформина и раком у пациентов с СД [57]. Авторы обнаружили отсутствие связи между лечением метформином и риском развития рака среди пациентов с СД, даже в группах с высокой суточной дозой (>1 г/сут) [57]. Тем не менее в этом исследовании могут быть некоторые неточности, обусловленные временными сдвигами. Поэтому для подтверждения этих результатов необходимы дальнейшие перспективные крупные популяционные когортные исследования.

## ВОЗМОЖНЫЙ ПРОТИВООПУХОЛЕВЫЙ МЕХАНИЗМ МЕТФОРМИНА

Как упоминалось выше, большинство исследований показывает, что метформин обладает противоопухолевым действием, однако механизм такого действия не был полностью выяснен. Считается, что метформин имеет прямые и косвенные противоопухолевые механизмы. Косвенные механизмы включают улучшение метаболизма глюкозы и липидов, ингибирование опухоль-ассоциированного воспаления и влияние на микроокружение опухоли. Прямое противоопухолевое действие обусловлено активацией АМФ-активируемой протеинкиназы (АМФК) и p53, а также ингибированием mTORC и ROS-JNK/c-Jun

## Улучшение метаболизма глюкозы и липидов

Гипергликемия может прямо или косвенно способствовать пролиферации, миграции и инвазии раковых клеток. В частности, высокий уровень глюкозы может усиливать передачу сигналов Wnt/β-катенина в раковых клетках, тем самым способствуя их пролиферации и росту [58]. Пациенты с СД2 также имеют высокий уровень инсулина и ИФР-1. Инсулин является основным регулятором клеточного метаболизма, а также фактором роста. Инсулин и ИФР-1 могут способствовать возникновению опухолей, стимулируя пролиферацию эпителиальных клеток [59]. Метформин может снижать уровень глюкозы в крови, улучшать резистентность к инсулину, а также снижать уровень ИФР-1 путем ингибирования разложения гликогена и глюконеогенеза печени, способствуя утилизации глюкозы в периферических тканях, увеличивая количество и сродство инсулиновых рецепторов, а также улучшая активность тирозинкиназы в мышечной и жировой тканях, что снижает риска возникновения и прогрессирования опухолей [60].

Принято считать, что нарушение липидного обмена связано с развитием неалкогольной жировой дистрофии печени, атеросклероза, ожирения и СД. В последние годы все большее число исследований показывает, что дислипидемия имеет связь с возникновением и развитием опухолей. Нарушенный липидный обмен может быть фактором риска возникновения таких заболеваний, как РМЖ, РП, РЛ и РЖ. Например, 27-гидроксихолестерин, метаболит холестерина с эстрогенной функцией, может связываться с эстрогенными рецепторами клеток РМЖ и способствовать их росту [61]. При повышении уровня липидов в крови увеличивается секреция лептина, а выработка адипонектина снижается. Лептин ингибирует апоптоз клеток РМЖ и непосредственно стимулирует экспрессию фактора роста эндотелиальных клеток сосудов, тем самым способствуя пролиферации опухолевых клеток и ангиогенезу [61]. Метформин способен избирательно повышать поглощение триглицеридов липопротеинами очень низкой плотности, а также окисление жирных кислот в бурой жировой ткани, тем самым нормализуя липидный обмен [53]. Метформин также активирует АМФК и ингибирует α-дикарбонил-опосредованную модификацию аполипопротеинов, устраняя дисфункцию липопротеинов высокой плотности и снижая модификацию липопротеинов низкой плотности, что приводит к улучшению транспорта холестерина и снижению накопления липидов в тканях, результатом чего является уменьшение риска возникновения и развития опухолей [53]. Кроме того, метформин ограничивает потребление калорий, что снижает поглощение липидов раковыми клетками из плазмы и тканевой жидкости.

## Ингибирование опухоль-ассоциированного воспаления

Хроническое воспаление связано с различными этапами развития опухолевого процесса, включая трансформацию клеток, пролиферацию, инвазию, ангиогенез и метастазирование [62].

Опухоль-ассоциированные макрофаги и активированный транскрипционный фактор NF-kB (ядерный фактор каппа B) стимулируют рост и пролиферацию опухолевых клеток, а также способствуют инвазии и метастазированию [53]. Метформин ингибирует воспаление, связанное с опухолевым процессом. Влияние метформина на опухоль-ассоциированное воспаление изучалось на модели мышей с трансгенным РПЖ. Результаты показали, что метформин останавливал инфильтрацию опухоль-ассоциированных макрофагов, ингибируя путь COX2/PGE2, тем самым прекращая прогрессирование РПЖ [53]. Обсервационное исследование показало, что лечение метформином в течение 12 нед может снижать концентрацию рецепторов 2-го типа фактора некроза опухоли (ФНО-Р2) в сыворотке крови пациентов с РМЖ и раком прямой кишки, в свою очередь, ФНО-α способствует росту опухолей путем активации фактора NF-kB через ФНО-Р2 [64]. Кроме того, метформин блокирует активность непосредственно NF-kB, что приводит к снижению секреции провоспалительных цитокинов [57]. Метформин избирательно снижает экспрессию рецептора интерлейкина-6 (ИЛ-6) на транскрипционном уровне через АМФК, мишень рапамицина (TOR — target of rapamycin) и микроРНК-34а, ингибирует ИЛ-6-зависимую активацию белка STAT3 (Signal transducer and activator of transcription 3), блокирует сигнальный путь STAT, снижает экспрессию Bcl-2 и циклина D1, а также повышает активность белка P21, тем самым ингибируя пролиферацию и индуцируя апоптоз клеток множественной миеломы [65].

## Влияние на микроокружение опухоли

Рак представляет собой сложную систему, в которой опухолевые клетки сосуществуют и взаимодействуют с различными типами незлокачественных клеток. Поэтому противоопухолевые препараты воздействуют не только на раковые клетки, но и наклетки микроокружения опухоли. В последние годы все большее количество исследований показывает, что микроокружение опухоли оказывает значительное влияние на опухолевые клетки и метаболизм опухоли [53]. Имеются данные, что метформин ингибирует рост, пролиферацию и метастазирование опухолей, воздействуя на опухоль-ассоциированное микроокружение, включая метаболическое состояние опухоли, ангиогенез, опухоль-ассоциированные фибробласты и иммунные клетки [66].

Основной особенностью метаболического перепрограммирования опухоли является аномальный окислительно-восстановительный метаболизм. Метформин может ингибировать окислительное фосфорилирование и снижать уровни АТФ в условиях низкой концентрации глюкозы, что может способствовать гибели опухолевых клеток [67]. Нарушение липидного обмена также является важным аспектом метаболического перепрограммирования опухоли. Повышенный обмен жирных кислот в опухолевых клетках индуцируется с целью удовлетворения энергетических потребностей для роста опухоли [68]. Синтаза жирных кислот является метаболическим онкогеном, который поддерживает рост и выживаемость опухолевых клеток; ее повышенная экспрессия наблюдается при многих видах рака [69]. Исследования показали, что метформин обладает способностью ингибировать адипогенез и адипоцитарно-опосредованную пролиферацию, а также метастазирование рака яичников, поэтому его применение рекомендуется для лечения рака яичников на ранних стадиях не только из-за его прямого противоопухолевого действия, но и благодаря его роли в изменении микроокружения опухоли [70].

Также было обнаружено, что метформин может ингибировать ангиогенез. Метформин снижает стабильность индуцируемого гипоксией фактора-1α (HIF-1α) в опухолевых клетках, а также экспрессию генов, нацеленных на HIF-1, включая фактор роста эндотелия сосудов-А (VEGF-A), что приводит к замедлению роста опухоли благодаря снижению количества новообразованных сосудов и их плотности [71]. Опухоль-ассоциированные фибробласты способствуют прогрессированию опухоли путем обеспечения опухоли ангиогенными факторами и питательными веществами, а также они принимают участие в инвазии опухолевых клеток [72]. Метформин ингибирует прогрессирование рака, непосредственно предотвращая передачу сигналов NF-kBот опухоль-ассоциированных фибробластов [73]. Опухоль-ассоциированные макрофаги включают провоспалительные и противоопухолевые макрофаги М1 (отвечают за уничтожение чужеродных агентов) и М2 (ускоряют регенерацию тканей). Исследования показали, что терапия метформином подавляет воспаление, опосредованное макрофагами. Было высказано предположение, что метформин-опосредованное увеличение внутриклеточной концентрации кислорода приводит к снижению выраженности гипоксии в некоторых опухолях, что повышает количество М1-макрофагов [74]. Кроме того, метформин может подавлять NF-kB, активируя АМФК, что в итоге приводит к преобладанию М1-макрофагов [75]. Раковые клетки способны снижать цитотоксичность лимфоцитов с помощью различных механизмов, наиболее известным из которых является сверхэкспрессия лиганда запрограммированной гибели клеток (PD-L1), что приводит к недостаточности цитотоксических свойств Т-клеток и, соответственно, к иммуносупрессии и повышенной устойчивости раковых клеток [76]. Терапия метформином может непосредственно увеличивать противоопухолевую активность лимфоцитов, снижать экспрессию PD-L1 на мембране раковых клеток, тем самым снижая их устойчивость [77].

Недавние исследования показали, что АМФК может контролировать дифференцировку стволовых клеток эпителия желудка. Метформин активирует АМФК и Круппел-подобный фактор 4, что снижает пролиферацию стволовых клеток эпителия у мышей, увеличивает количество париетальных клеток желудка путем активации АМФК и пролифератора пероксисом гамма, тем самым повышая секрецию желудочного сока и косвенно снижая риск развития РЖ [78].

## Прямые механизмы

Прямой противоопухолевый эффект метформина связан с активацией АМФК. АМФК — это клеточная протеинкиназа, которая контролирует энергетический баланс клетки [53]. В условиях метаболического стресса, например при гипоксии или дефиците глюкозы, соотношение АМФ/АТФ увеличивается, что приводит к активации АМФК. TOR является основным регулятором роста и пролиферации клеток. АМФК может ингибировать биосинтез и рост клеток путем блокирования сигнального комплекса TORC1 [79]. C-myc является основным регулятором метаболизма раковых клеток и влияет на гликолиз и катаболизм глутамина. Регуляция экспрессии микроРНК лежит в основе противоопухолевого действия метформина. Он активирует цепь АМФК-DICER-микроРНК и оказывает регулирующее воздействие на цепь DICER-микроРНК, что приводит к снижению уровней c-myc, HIF-1α и IRS2 (insulin receptor substrate 2), в конечном итоге нарушая метаболизм опухолевых клеток и ингибируя развитие опухоли [80].

Фосфорилирование АМФК индуцирует остановку клеточного цикла путем прямого фосфорилирования p53, который имеет много биологических функций, включая регулирование роста клеток, их развития и старения [81]. Метформин способен ингибировать пролиферацию и индуцировать апоптоз клеток рака шейки матки, активируя АМФК и другие сигнальные пути, тем самым снижая экспрессию циклина D1 и повышая экспрессию p53 [81].

Метформин также ингибирует развитие и прогрессирование опухолей независимо от сигнального пути АМФК. Метформин индуцирует апоптоз и аутофагию в клетках ESCC (Esophageal squamous cell carcinoma) путем инактивации STAT3 и сдерживания экспрессии Bcl-2 [82], а также индуцирует апоптотические пути в клетках рака надпочечников и поджелудочной железы путем активации каспазы-3 [83]. Кроме того, метформин индуцирует митохондриальный апоптоз в раковых клетках яичников путем повышения уровня белка Bax и расщепленной каспазы-3 [84]. Метформин также ингибирует рост и инвазию клеток гепатоцеллюлярной карциномы через путь PI3K/Akt/mTOR, а также индуцирует апоптоз и аутофагию [85]. Недавние исследования показали, что метформин индуцирует остановку клеточного цикла и апоптоз в клетках остеосаркомы человека через каскад ROS-JNK/c-Jun [86].

## ЗАКЛЮЧЕНИЕ

Метформин является противодиабетическим препаратом 1-й линии при СД2; его молекулярной мишенью является АМФК, которая участвует во многих метаболических процессах. Метформин не только способствует снижению уровня глюкозы в крови и улучшает чувствительность к инсулину, но также ингибирует липолиз и снижает сердечно-сосудистый риск у пациентов с СД2. В последние годы было доказано, что метформин замедляет процесс старения, стимулирует рост волос, устраняет когнитивные нарушения, а также обладает противоопухолевым эффектом.

На сегодняшний день большинство клинических исследований доказало, что лечение метформином может снизить риск развития рака и увеличить выживаемость больных раком. Тем не менее преимущества метформина при некоторых видах рака неясны, т.к. иногда он может ускорять прогрессирование заболевания. Из-за своей полярности метформин проникает в клетки через мембранные транспортеры. Органические транспортеры катионов (ОТК) играют важную роль для проникновения метформина в клетки, в свою очередь, опухолевые клетки могут экспрессировать ОТК1. Однако различные виды опухолей могут иметь разные уровни экспрессии ОТК1, снижение экспрессии ОТК1 в опухолевых клетках будет уменьшать поглощение метформина, тем самым влияя на его эффективную концентрацию; этот фактор может объяснить различное влияние метформина на опухоли.

В настоящее время протективный эффект метформина доказан при многих видах опухолей, включая РЛ, РЖ, колоректальный рак и РП, его можно применять в качестве адъювантной терапии. Тем не менее влияние метформина на РМЖ и РПЖ все еще обсуждается, его применение нежелательно у пациентов без СД. Необходимо проведение дополнительных проспективных исследований с целью оценки влияния метформина на различные виды рака. Кроме того, дальнейшее исследование экспрессии ОТК1 в различных опухолевых клетках может способствовать лучшему пониманию противоопухолевого действия метформина.

Большинство фундаментальных исследований показало, что метформин ингибирует рост опухолевых клеток и способствует клеточному апоптозу, в то время как клинические исследования показывают противоречивые результаты. Такое несоответствие можно объяснить разницей в концентрации метформина между фундаментальными и клиническими исследованиями. Максимальная суточная доза метформина для пациентов с СД2 составляет 2500 мг/сут, а доза, использованная вфундаментальных исследованиях, была намного выше. Метформин непосредственно активирует сигнальный путь АМФК, ингибирует выработку активных форм кислорода, индуцирует активацию mTORC1, ингибирует циклин D1, что приводит к снижению риска возникновения и развития злокачественных новообразований. Кроме того, метформин косвенно ингибирует рост опухоли, пролиферацию, инвазию и метастазирование путем снижения концентрации глюкозы в крови, улучшения чувствительности к инсулину, а также путем уменьшения воспаления и влияния на микроокружение опухоли. Гликолиз играет важную роль в энергетическом обмене опухолей, а метформин способен оказывать на него ингибирующее влияние. В настоящее время исследования механизма противоопухолевых эффектов метформина становятся все более обширными и углубленными, однако по-прежнему остаются некоторые противоречия.

## ДОПОЛНИТЕЛЬНАЯ ИНФОРМАЦИЯ

Источники финансирования. Работа выполнена по инициативе авторов без привлечения финансирования.

Конфликт интересов. Авторы декларируют отсутствие явных и потенциальных конфликтов интересов, связанных с содержанием настоящей статьи.

Участие авторов. Кузнецов К.О. — разработка концепции и дизайна исследования, получение и анализ данных, интерпретация результатов; Сафина Э.Р. — разработка дизайна исследования, написание статьи; Гаймакова Д.В. — анализ данных, написание статьи; Фролова Я.С. — интерпретация результатов, написание статьи; Оганесян И.Ю. — получение и анализ данных, редактирование статьи; Садертдинова А.Г. — интерпретация результатов, редактирование статьи; Назмиева К.А. — анализ данных, редактирование статьи; Исламгулов А.Х. — получение данных, редактирование статьи; Каримова А.Р. — получение данных, редактирование статьи; Галимова А.М. — получение данных, редактирование статьи; Ризванова Э.В. — получение данных, редактирование статьи. Все авторы внесли равный вклад в написание статьи и одобрили ее финальную версию перед публикацией, выразили согласие нести ответственность за все аспекты работы, подразумевающую надлежащее изучение и решение вопросов, связанных с точностью или добросовестностью любой части работы.
